# Molecular and Cellular Mechanisms of Apoptosis during Dissociated Spermatogenesis

**DOI:** 10.3389/fphys.2017.00188

**Published:** 2017-03-29

**Authors:** Tengfei Liu, Lingling Wang, Hong Chen, Yufei Huang, Ping Yang, Nisar Ahmed, Taozhi Wang, Yi Liu, Qiusheng Chen

**Affiliations:** Laboratory of Animal Cell Biology and Embryology, College of Veterinary Medicine, Nanjing Agricultural UniversityNanjing, China

**Keywords:** dissociated spermatogenesis, testes, apoptosis, Chinese soft-shelled turtle (*Pelodiscus sinensis*), digital gene expression

## Abstract

Apoptosis is a tightly controlled process by which tissues eliminate unwanted cells. Spontaneous germ cell apoptosis in testis has been broadly investigated in mammals that have an associated spermatogenesis pattern. However, the mechanism of germ cell apoptosis in seasonally breeding reptiles following a dissociated spermatogenesis has remained enigmatic. In the present study, morphological evidence has clearly confirmed the dissociated spermatogenesis pattern in *Pelodiscus sinensis*. TUNEL and TEM analyses presented dynamic changes and ultrastructural characteristics of apoptotic germ cells during seasonal spermatogenesis, implying that apoptosis might be one of the key mechanisms to clear degraded germ cells. Furthermore, using RNA-Seq and digital gene expression (DGE) profiling, a large number of apoptosis-related differentially expressed genes (DEGs) at different phases of spermatogenesis were identified and characterized in the testis. DGE and RT-qPCR analysis revealed that the critical anti-apoptosis genes, such as *Bcl-2, BAG1*, and *BAG5*, showed up-regulated patterns during intermediate and late spermatogenesis. Moreover, the increases in mitochondrial transmembrane potential in July and October were detected by JC-1 staining. Notably, the low protein levels of pro-apoptotic cleaved caspase-3 and CytC in cytoplasm were detected by immunohistochemistry and western blot analyses, indicating that the CytC-Caspase model might be responsible for the effects of germ cell apoptosis on seasonal spermatogenesis. These results facilitate understanding the regulatory mechanisms of apoptosis during spermatogenesis and uncovering the biological process of the dissociated spermatogenesis system in reptiles.

## Introduction

Spermatogenesis is a highly organized and complex process that is divided into the mitotic division of spermatogonia, spermatocytic meiosis and the production of mature spermatozoa in the testes of male vertebrates (de Kretser et al., 1998). Reptiles, occupying a strategic position among terrestrial vertebrates, possess tubular testes in which multiple generations of germ cells develop within the seminiferous epithelia (Gribbins, 2011). It is well-known that many mammals have an associated reproductive pattern. In some seasonally breeding reptilian species, however, spermatogenesis is highly seasonal and not coupled in time with male mating behavior, which is called a dissociated reproductive pattern (Gribbins et al., 2006; Rheubert et al., 2009a; Clesson et al., 2013). These reptiles have germ cell cohorts that develop together as a population and are typically released into centralized lumina in one unified spermiation event at the end of spermatogenesis. Currently, the episodic germ cell development strategy has been extensively studied in numerous temperate chelonians in which the principal costs of spermatogenesis arise before the breeding season and cease during the breeding season (Gribbins et al., 2003; Sousa et al., 2014; Lancaster et al., 2015). Although the dissociated spermatogenesis system has been widely described and the transitional and evolutionarily significance has also been reported in many turtle species, the mechanisms underlying dissociated spermatogenesis have not been fully elucidated.

Considerable studies have suggested that the spontaneous death of germ cells in testis is conspicuous during the normal spermatogenesis of many species (Weinbauer et al., 2001; Shaha and Mishra, 2010). It has been shown that apoptosis plays an essential role in the potential spermatozoa output and the guaranteed of semen quality (Schaller et al., 2008; Mahfouz et al., 2009). Furthermore, apoptosis has been reported to contribute to germ cell degeneration and seasonal testicular regression in many species, including crow, argenteus, brown hare, and European starlings (Young et al., 2001; Strbenc et al., 2003; Islam et al., 2012; Domingos et al., 2013). In mammals, the apoptosis of Sertoli cells is found to directly govern the formation of seminiferous tubule lumen (Walczak-Jedrzejowska et al., 2007). Recent studies have proposed that an early and massive wave of apoptosis initiates during the first round of spermatogenesis to maintain the appropriate ratio between germ cells and Sertoli cells (Rodriguez et al., 1997). Other studies, however, suggest that the occurrence of apoptosis in testis will create a certain amount of problems influencing normal spermatogenesis. For instance, in patients with cryptorchidism, the proportion of apoptotic germ cells was markedly higher than in the normal group, whereas the disturbance of spermatogenic cell apoptosis could improve male infertility (Oosterhuis et al., 2000; Kwon et al., 2004; Weikert et al., 2005). Despite the intricate relationship between apoptosis and normal spermatogenesis having attracted the attention of many researchers, studies on the regulation of apoptosis determining the biological process of dissociated spermatogenesis system are sparse, especially in temperate freshwater turtles.

The Chinese soft-shelled turtle, *Pelodiscus sinensis*, belonging to ectothermic amniotic reptiles, has high nutritional and pharmaceutical value and is widely raised in freshwater lakes of Asian countries such as China, Japan, and Korea. *P. sinensis*, like most temperate freshwater turtles, has a typical pattern of episodic germ cell development (Zhang et al., 2007; Ullah et al., 2014). The spermatogenesis of *P. sinensis* initiates in the spring and continues through summer and fall, with one massive release of mature sperm in early November, while it is inactive throughout the rest of the year (December to next April, the hibernation season; Nisar et al., 2016). Thus, the Chinese soft-shelled turtle can be used as a potential model organism to explore the molecular mechanisms of the dissociated spermatogenesis system. Furthermore, the draft genome sequences of *P. sinensis* have been published and provide an invaluable resource and favorable chance for research into the mechanisms of spermatogenesis in the Chinese soft-shelled turtle (Wang et al., 2013).

Our previous studies have systematically investigated the ultrastructural and cellular particularities of spermatogenesis in *P. sinensis* (Zhang et al., 2007, 2008). Moreover, biochemical, and molecular genetic studies in female *P. sinensis* have discovered a number of apoptosis-related genes in which some critical genes were also demonstrated to be involved in spermatogenesis (Zhang et al., 2013; Liu et al., 2016b). Recently, the RNA sequencing (RNA-Seq) technique serves as an accurate and appropriate approach when combined with digital gene expression (DGE) tag profiling, and it has been successfully used for the expression analysis of transcriptional changes in some reptile species, including brown treesnake (*Boiga irregularis*), *anolis* lizard (*Anolis allogus*), alligator (*Alligator mississippiensis*), and western painted turtle (*Chrysemys picta bellii*; Mcgivern et al., 2014; Akashi et al., 2016; Jiang et al., 2016; Yatsu et al., 2016). Nevertheless, there is no report concerning the global expression profiling of apoptosis-related genes during the process of dissociated spermatogenesis, and the mechanism underlying spermatogenesis was largely unexplored in *P. sinensis*.

In this study, the characteristics of spermatogenesis and variation tendencies of apoptosis during spermatogenesis in *P. sinensis* were studied by using morphological evidence. To investigate the integrated and comprehensive characterization of the genes regulating apoptosis and dissociated spermatogenesis, RNA-Seq and DGE profiling using the *P. sinensis* testis in different stages of spermatogenesis (April, July, and October) were performed with the Illumina HiSeq 2500 platform. The objectives were to identify the differentially expressed genes (DEGs) involved in the regulation of apoptosis and spermatogenesis and to provide a more complete evaluation of apoptosis influencing dissociated spermatogenesis at the molecular level. The expression patterns of DEGs related to spermatogenesis were detected by RT-qPCR analysis. Furthermore, immunohistochemistry (IHC) and western blot analysis were further implemented to validate the protein level variations of critical genes. The outcomes of this study provide significant insights toward the regulatory roles of apoptosis in the spermatogenesis of *P. sinensis* and facilitate uncovering the molecular regulatory mechanisms underlying the dissociated spermatogenesis system in reptiles.

## Materials and methods

### Ethics statement

In this study, all experimental procedures and animal care were conducted according to the recommendations of the Guide of the Nanjing Agriculture University Animal Care and Use Committee (Nanjing Agriculture University, Jiangsu, China). The protocol was approved by the Science and Technology Agency of Jiangsu Province under permit NO. SYXK (SU) 2010-0005, and all efforts were made to minimize animal suffering. The field studies did not involve any endangered or protected species.

### Animals

Adult male Chinese soft-shelled turtles *P. sinensis* (with an average weight and plastron length of 1.16 ± 0.12 kg and 17.45 ± 1.63 cm, respectively) were collected from Yangcheng Lake in Suzhou (31°N, 120°E), southeastern China, during the months of April (*n* = 10), July (*n* = 10), and October (*n* = 10) in 2015. The turtles were rendered comatose by using an intraperitoneal injection of sodium pentobarbital (20 mg/kg) and were subsequently sacrificed through cervical dislocation. One side of the testis sample of each turtle was collected and fixed for light and transmission electron microscopy. The other side of the testis was ground in liquid nitrogen immediately and kept at −80°C for gene expression analysis.

### Haematoxylin-eosin (H&E) staining

The tissues were fixed in 20% paraformaldehyde solution for 48 h and embedded in paraffin. Paraffin-embedded blocks were cut to 6 μm slices using a microtome device (Leica, RM 2245, Germany). The sections were dewaxed and stained with a haematoxylin-eosin staining method that was previously described by Liu et al. (2016a). At this point, the samples were observed under a light microscope (Olympus DP73).

### TdT-mediated dUTP nick-end labeling (TUNEL) assay

The TUNEL assay was performed using an *in situ* cell Apoptosis Detection Kit (S7100, Millipore, Billerica, MA, USA) according to the instructions of the manufacturer. The paraffin embedded tissue sections were deparaffinized and rehydrated and then treated with proteinase K at 20 μg/mL for 20 min at room temperature. Sections were incubated with reaction buffer containing TdT enzyme and at 37°C for 1 h. After washing with stop/wash buffer, sections were treated with anti-digoxigenin conjugate for 30 min at room temperature and subsequently developed color in peroxidase substrate. After mounting the TUNEL-positive cells, the nuclei were counterstained with haematoxylin. Negative controls were obtained by incubating sections without TdT enzyme. The percentage of apoptotic cells was calculated as a ratio of the number of TUNEL-positive cells to the total number of cells. Eight fields per section were randomly examined at ×400 magnification under a light microscope (Olympus DP73).

### Transmission electron microscopy (TEM)

Testicular parenchyma was fixed with 2.5% glutaraldehyde in 0.2 M PBS (PH 7.4) at 4°C for 48 h. The tissue was then post-fixed in 1% osmium tetroxide for 2 h at 4°C. After dehydration in a graded series of alcohol (75, 85, 95, and 100%, each for 10 min), the samples were embedded in epoxy resin. Ultrathin sections, 50 nm thick, were mounted on copper grids, stained with uranyl acetate and lead citrate, and examined with a Hitachi HT7700 transmission electron microscope at 80 kV.

### RNA extraction, library construction, and illumina sequencing

The testis samples from 10 turtles in April, July, and October were pooled in equimolar quantity for RNA extraction and library construction. Total RNA was isolated using TRIzol reagent (Life Technologies, Carlsbad, CA, USA) and treated with RNase-free DNaseI (TaKaRa, Otsu, Japan) to remove genomic DNA contamination. The standard Illumina protocol was followed to develop RNA-Seq libraries. Three cDNA libraries of *P. sinensis* named MT_1, MT_2, and MT_3 were constructed separately from the tissues in April, July, and October, and sequenced according to a previously reported method (Liu et al., 2016b). The Illumina HiSeq 2500 platform was used for sequencing, and 150-bp paired-end reads were generated. All raw-sequence read data were deposited in the NCBI Sequence Read Archive (SRA, http://www.ncbi.nlm.nih.gov/Traces/sra/) with accession numbers of SRX2351846 (MT_1), SRX2352135 (MT_2), and SRX2352136 (MT_3).

### Data processing and analysis of DEGs

Raw reads were pre-processed to remove both the reads containing adapter and ploy-N and low-quality sequences (*Q* < 20). Clean reads were obtained and mapped to the Chinese soft-shelled turtle genome sequence (Wang et al., 2013) using the program TopHat v2.0.12 with only 1 bp mismatch allowed. The read numbers of each gene were calculated using HTSeq v0.6.1 and adjusted by the edgeR program package through one scaling normalized factor. The level of gene expression was normalized using the fragments per kilobase of transcript sequence per millions (FPKM) method. A rigorous algorithm described previously was used to further identify DEGs among different libraries (Audic and Claverie, 1997). Genes with the absolute value of |log_2_ Ratio| ≥ 1, *P* < 0.05 and False Discovery Rate (FDR) ≤ 0.001 were determined to be significantly differentially expressed. The gene expression patterns were analyzed, and genes were clustered using Cluster 3.0 software and Java Treeview software.

### Annotation of DEGs

All of the DEGs were subjected to the Gene Ontology (GO) and Kyoto Encyclopedia of Genes and Genomes (KEGG) annotations. The gene sequences were searched against the NCBI Nr database with BLAST. GO annotations and functional classifications of DEGs were obtained using the Blast2GO program and WEGO software. The GOseq R package was used to perform GO enrichment analysis of the DEGs. KEGG pathway assignments were carried out using KOBAS software against the KEGG database (Kanehisa et al., 2008). *P* ≤ 0.05 was used as the threshold to identify the significantly enriched GO terms and pathways.

### Quantitative real-time PCR (RT-qPCR) validation of RNA-Seq data

Total RNA used for RT-qPCR analysis was prepared from the *P. sinensis* testes in April, July, and October with three biological replicates. The cDNA was synthesized using the SuperScript First-Strand Synthesis System (Invitrogen, Carlsbad, CA, USA). Primer sequences were designed by using Beacon Designer 7.0 (Premier Biosoft International, USA) software (Supplementary Table 1). The RT-qPCR analysis with three technological replications was performed on an iCycler Real-Time PCR Detection System (Bio-Rad, USA) according to a previous report (Kim et al., 2013). The β*-Actin* gene was used as the internal control. The relative expression levels of the genes were calculated using the delta-delta-CT method.

### Isolation of mitochondrial fraction

Testis mitochondrial fractions were prepared as described previously (Latchoumycandane et al., 2002). Briefly, fresh *P. sinensis* testis was cut into small cubes with scissors and homogenized using a glass-to-glass homogenizer (30–50 times). The homogenate was centrifuged at 800 × g for 10 min and maintained at 4°C. The supernatant was discarded, and the pellets were suspended in 200 μL of 12% Percoll, carefully laid on 1 mL of 24% Percoll and centrifuged for 10 min at 15,000 × g (4°C). The purified mitochondrial fraction was visible at the bottom of the tube. To remove residual Percoll, the mitochondrial fraction was diluted in ice-cold isolation medium (250 mmol/L sucrose, 10 mmol/L HEPES-KOH, pH 7.2) and washed three times by centrifugation at 10,000 × g for 10 min. The protein concentration of the mitochondrial fraction was determined by the method of Lowry et al. (1951).

### Measurement of mitochondrial transmembrane potential

The mitochondrial transmembrane potential was determined with the dye 5,5′,6,6′-tetrachloro-1,1′,3,3′-tetraethyl benzimidazol carbocyanine iodide (JC-1; ab113850, Abcam Inc., Cambridge, MA, USA). To decrease the probability of pitfalls due to uncoupling of mitochondria, the mitochondrial transmembrane potential was analyzed within the first hour after mitochondrial isolation. The isolated mitochondria (0.5 mg/mL) were incubated at 37°C for 30 min in sucrose buffer (220 mmol/L mannitol, 75 mmol/L sucrose, 10 mmol/L HEPES-KOH pH 7.4, 1 mmol/L EDTA, 0.5% BSA) in the presence of JC-1 (1 μg/mL). At the end of the incubation, the dye loaded mitochondria were collected by centrifugation, rinsed three times for 2 min each with dilution buffer (from Reagent kit) to remove the excess dye and then resuspended in sucrose buffer in appropriate dilution. The fluorescence of the JC-1 monomer (green) and aggregate (red) was read in a fluorescence plate-reader (BMG Labtech, Offenburg, Germany) with excitation/emission settings at 485/535 and 560/595 nm, respectively. The ratio of JC-1 aggregate to monomer (the 595:535 ratio) was calculated. A decrease in this ratio was interpreted as a decrease in the mitochondrial transmembrane potential, whereas an increase in the ratio was interpreted as a gain in the mitochondrial transmembrane potential. The experiments were repeated at least three times.

### Immunohistochemistry (IHC)

IHC was performed according to a previously reported method (Li et al., 2015). Briefly, testicular sections were dewaxed with xylene and dehydrated in serially graded ethanol solutions. The endogenous peroxidase activity was quenched with 3% H_2_O_2_ in distilled water. The sections were applied with anti-Bcl-2 antibody (ab196495, Abcam Inc., Cambridge, MA, USA; 1:50), anti-Cleaved caspase-3 antibody (AB3623, Merck Millipore, Billerica, MA, USA; 1:20), and anti-Cytochrome C (CytC) antibody (ab13575, Abcam Inc., Cambridge, MA, USA; 1:100) separately and then incubated overnight at 4°C. As a negative control, some sections were reacted with PBS instead of the specific antibodies. After reacting with a biotin-conjugated secondary antibody (ab6720, Abcam Inc., Cambridge, MA, USA; 1:1,000) for 1 h at room temperature, the sections were stained using the FAST DAB Peroxidase Substrate (Sigma, St Louis, MO, USA) and counterstained with haematoxylin for 10 s. The slides were then dehydrated and mounted for observation.

### Western blot analysis

Western blot was performed using equal amounts of protein (50 μg/lane) from the testis. For CytC, the cytosolic and mitochondrial proteins were isolated from the testes according to the protocols of the Tissue Mitochondria Isolation Kit (Pierce Biotechnology, Rockford, IL, USA). The primary antibodies to Bcl-2 (ab196495, Abcam Inc., Cambridge, MA, USA; 1:1,000), Cleaved caspase-3 (AB3623, Merck Millipore, Billerica, MA, USA; 1:200), CytC (ab13575, Abcam Inc., Cambridge, MA, USA; 1:1,000), COX IV (ab16056, Abcam Inc., Cambridge, MA, USA; 1:1,000), and β-Actin (4970, Cell Signaling Technology, Beverly, MA, USA; 1:1,000) were added and incubated at 4°C overnight. The immunoreactive bands were quantified using Quantity One software (Bio-Rad Laboratories). The validity of the antibodies in *P. sinensis* was determined by negative and positive control analysis. The protein from mouse testis was the positive control. The negative controls were obtained by exchanging primary antibody with 0.01 M PBS.

### Statistical analysis

Data are expressed as the mean ± standard error of the mean (SEM). Statistical analysis was conducted with SPSS 16.0 software (SPSS, Inc., Chicago, IL, USA), and significance was assessed by one-way ANOVA. The level of significance was set at *P* < 0.05.

## Results

### Spermatogenic and seminiferous epithelium cycle

**Figure 1:**
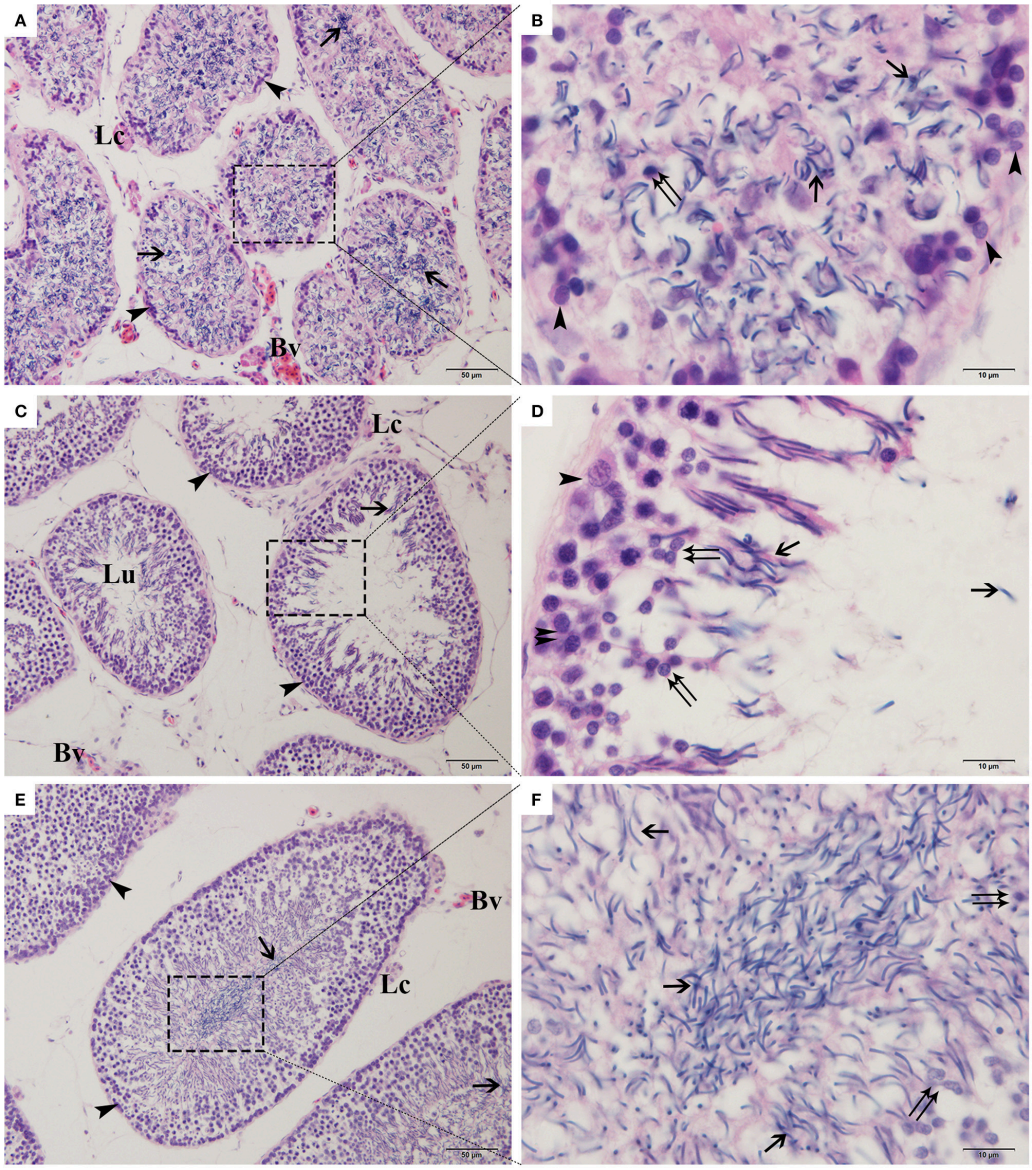
**Transverse views of seminiferous tubules from ***P. sinensis*** testes collected in April (A,B)**, July **(C,D)**, and October **(E,F)**. Arrowhead, spermatogonia; Double arrowhead, spermatocyte; Double arrow, spermatid; Arrow, spermatozoa; Lc, Leydig cell; Bv, blood vessel; Lu, lumen. Scale bar = 50 μm **(A,C,E)** and 10 μm **(B,D,F)**.

In the testes of *P. sinensis*, the seminiferous tubules are lined along the seminiferous epithelium, which rested on a basement membrane and surrounded a centrally located lumen. In April, the spermatogenically quiescent phase, the seminiferous epithelium contained a few layers of germ cells, typical spermatogonia and residual germ cells (most were spermatids and spermatozoa; Figures 1A,B). It was common to see that the elongated spermatids were arranged in the sperm columns from the basal to the adluminal compartment in July (intermediate spermatogenesis; Figures 1C,D). In October (late spermatogenesis), the majority of cells in the seminiferous epithelium were elongated spermatids and mature spermatozoa that located in the lumen (Figures 1E,F). These observations demonstrated the highly seasonal model of spermatogenesis in *P. sinensis*, and thus, the testis samples in April, July, and October were used as representative samples to study dissociated spermatogenesis in this study.

### Detection of testicular germ cell apoptosis by TUNEL and TEM

**Figure 2:**
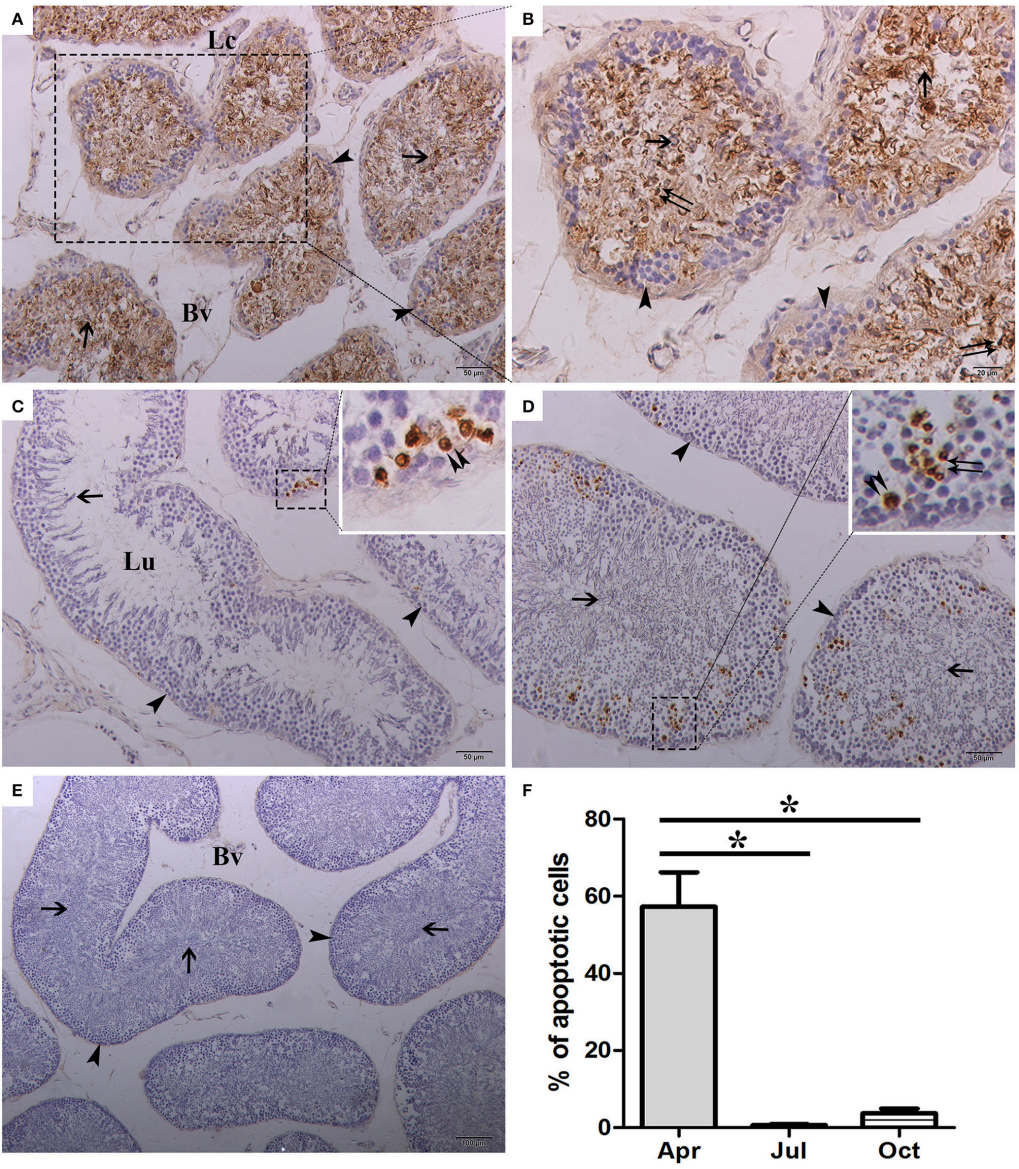
**Representative photographs of TUNEL staining in April (A,B)**, July **(C)**, and October **(D)** as well as negative control **(E)** of the testis and the counting of TUNEL-positive cells **(F)**. Arrowhead, spermatogonia; Double arrowhead, spermatocyte; Double arrow, spermatid; Arrow, spermatozoa; Lc, Leydig cell; Bv, blood vessel; Lu, lumen. Scale bar = 100 μm **(E)**, 50 μm **(A,C,D)** and 20 μm **(B)**. The values (^*^) obtained in April were significantly higher (*P* < 0.05) than those obtained in July and October.

To evaluate the relationship between apoptosis and spermatogenesis in *P. sinensis*, the apoptosis of testicular cells in different stages of spermatogenesis was investigated by *in situ* 3′-OH end tailing of fragmented DNA. As a result, a large number of apoptotic cells were observed in April. These TUNEL-positive cells were mainly spermatids and spermatozoa, whereas positive staining was seldom observed in the spermatogonia that were near the basal portion of the seminiferous epithelium (Figures 2A,B). In July, the number of apoptotic cells decreased dramatically, and most of the observed positive cells were spermatocytes that were undergoing apoptosis occasionally (Figure 2C). In October, the TUNEL-positive cells were slightly increased compared with July, and the apoptotic cells were mainly distributed among the spermatocytes and spermatids (Figure 2D). No labeling was seen in the negative control sections (Figure 2E). Moreover, statistical analysis showed that the rate of apoptosis was significantly higher in April than in July and October (*P* < 0.05; Figure 2F). These results revealed that apoptosis is relatively high during the spermatogenically quiescent phase in *P. sinensis*.

**Figure 3:**
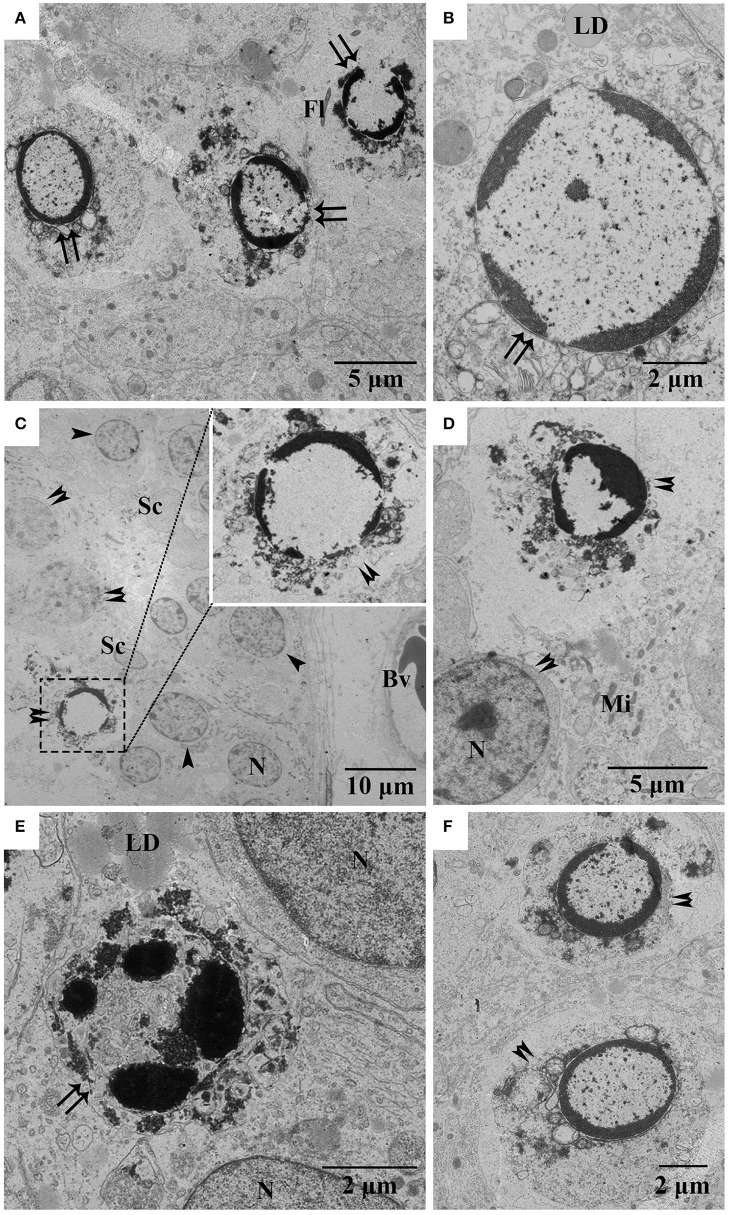
**Electron micrographs of various germ cell types undergoing apoptosis during spermatogenesis. (A,B)** Apoptotic spermatids in April, **(C,D)** apoptotic spermatocytes in July, **(E)** apoptotic spermatids in October, **(F)** apoptotic spermatocytes in October. Arrowhead, spermatogonia; Double arrowhead, spermatocyte; Double arrow, spermatid; Fl, flagellum; LD, lipid droplet; Bv, blood vessel; Mi, mitochondria; N, nucleus. Scale bar = 10 μm **(C)**, 5 μm **(A,D)**, and 2 μm **(B,E,F)**.

To further detect the occurrence of apoptosis in the testis of *P. sinensis*, we observed the ultrastructural morphology of the testis by TEM in April, July and October. In April, the apoptotic spermatids exhibiting the typical features of apoptotic cells such as membrane blebbing and chromatin condensation were observed by TEM (Figures 3A,B). In July, a shape change in the condensed nuclear chromatin that was mostly the half-moon type was observed in spermatocytes (Figures 3C,D). Nevertheless, the apoptotic germ cells, including both the spermatocytes and spermatids, were detected in October (Figures 3E,F). The events of apoptosis in the different stages of spermatogenesis observed by TEM were consistent with the results of TUNEL analysis.

### RNA-Seq analysis and spermatogenesis-related DEGs identification

**Figure 4:**
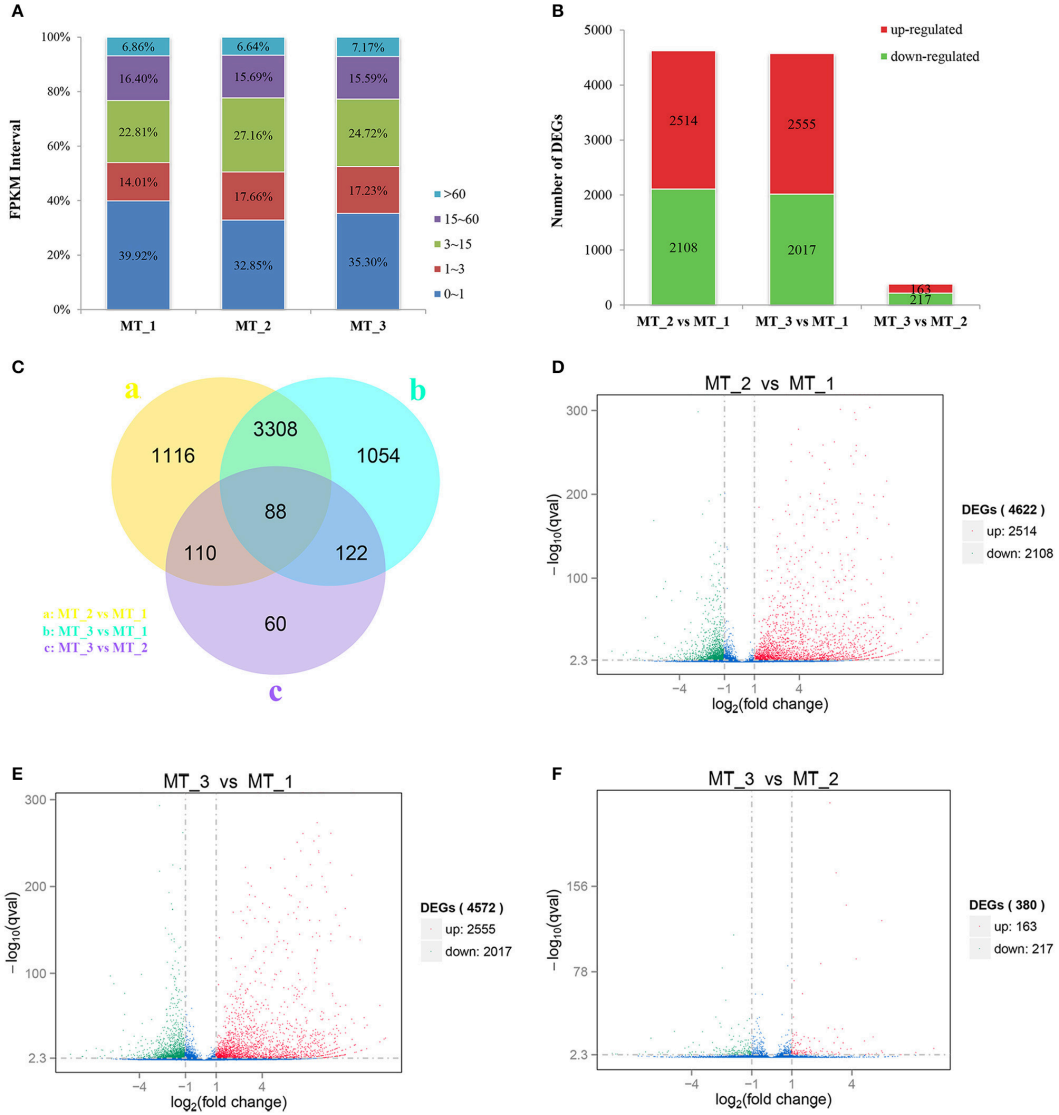
**Distribution of genes with differential expression in MT_1, MT_2, and MT_3 libraries. (A)** Statistics of FPKM-value and percentage of the identified DEGs in three libraries. **(B)** Number of DEGs in the three libraries. **(C)** Unique and common DEGs among the three libraries. **(D,E,F)** Volcano plot of gene expression differences in pairs of MT_2 vs. MT_1, MT_3 vs. MT_1, and MT_3 vs. MT_2, respectively.

In view of the differences in the morphological features at different stages of spermatogenesis, the testes of *P. sinensis* in April, July and October were further chosen for RNA-Seq analysis to identify the DEGs involved in the spermatogenesis. Three cDNA libraries named MT_1, MT_2, and MT_3 were prepared and sequenced with the Illumina HiSeq 2500 platform. A total of 62,840,880 (MT_1), 45,060,196 (MT_2), and 43,378,150 (MT_3) clean reads were obtained from these three libraries (Supplementary Table 2), with 71.31, 73.14, and 73.45% of the reads being successfully mapped to the Chinese soft-shelled turtle reference genome, respectively. To examine the expression of each gene during spermatogenesis of *P. sinensis*, their read counts were determined and analyzed using the FPKM method (Figure 4A). In total, 4,622, 4,572, and 380 significant DEGs were identified in the comparisons of MT_2 vs. MT_1, MT_3 vs. MT_1, and MT_3 vs. MT_2 (Figure 4B), respectively. The unique and common expressions of the genes among these three libraries are shown in Figure 4C. Of these DEGs, 2514 up- and 2108 down-regulated genes were identified between the MT_2 and MT_1 libraries (Figure 4D), and 2555 up- and 2017 down-regulated genes were detected between the MT_3 and MT_1 libraries (Figure 4E). It is obvious that the majority of DEGs were assigned to the pairs of MT_2 vs. MT_1 and MT_3 vs. MT_1, while most of the gene levels have no significant difference between the MT_2 and MT_3 libraries, which obtained a smaller number of DEGs (Figure 4F). The results imply that these identified DEGs between the pairs of April vs. July (MT_2 vs. MT_1), and April vs. October (MT_3 vs. MT_1) may be directly related to the spermatogenesis regulation of *P. sinensis*.

### Functional annotation and enrichment analysis of DEGs

The functional annotation and enrichment analysis of DEGs was performed to assign them with GO terms and KEGG pathways. Three main GO categories included molecular function, cellular component, and biological process. Using *P* ≤ 0.05 as the threshold value, a total of 177 and 199 GO terms were significantly enriched in the MT_2 vs. MT_1 and MT_3 vs. MT_1 comparisons (Supplementary Table 3), respectively, while no terms were enriched for the DEGs between MT_3 and MT_2. Notably, several enriched GO terms related to sperm and spermatogenesis were found, such as “sperm part” (GO: 0097223), “sperm flagellum” (GO: 0036126), “spermatogenesis” (GO: 0007283), “spermatid development” (GO: 0007286), “spermatid differentiation” (GO: 0048515), and “sperm motility” (GO: 0030317; Supplementary Table 3). DEGs involved in these critical GO categories might play vital roles in the process of *P. sinensis* spermatogenesis. Moreover, pathway enrichment analysis revealed that 46, 39, and 12 significantly enriched pathways were identified in the comparisons of MT_2 vs. MT_1, MT_3 vs. MT_1, and MT_3 vs. MT_2 (Supplementary Table 4), respectively. The most DEGs were involved in the pathways of “Metabolic pathways” (pss01100), “Ribosome” (pss03010), and “MAPK signaling pathway” (pss04010), both in MT_2 vs. MT_1 and MT_3 vs. MT_1.

### Identification of apoptosis-related DEGs during spermatogenesis

Pathway-based analysis identified a number of DEGs involved in various biological processes and signaling pathways. Apoptosis is a physiological programmed cell death process and plays vital roles during spermatogenesis in *P. sinensis*. In this study, a total of 144 DEGs were identified and associated with apoptosis regulation during spermatogenesis (Supplementary Table 5). Remarkably, 42 DEGs were found to be involved in the “Apoptosis” pathway (pss04210) and “p53 signaling pathway” (pss04115; Table 1). Some critical genes, including anti- and pro-apoptosis genes, showed significantly differential expression. For instance, *Bcl-2* and *BCL2L1* as anti-apoptosis genes were up-regulated in MT_2 and MT_3 libraries compared with in MT_1, while *TRADD, FADD, PTEN*, and *AIFM1* were down-regulated. Moreover, several genes, such as *PIK3CD, PPP3CC, CFLAR, PIK3R1*, and *PPP3CA*, exhibited uniform expression patterns in all three comparisons of MT_2 vs. MT_1, MT_3 vs. MT_1, and MT_3 vs. MT_2 (Table 1). Some genes implicated in the process of p53 signal transduction have been reported to participate in the regulation of apoptosis. For example, several transcripts encoding *CASP3, FAS, PIDD, PERP*, and *CytC* were down-regulated during spermatogenesis (Table 1), indicating that these DEGs were involved in apoptosis regulation and may play roles in the process of *P. sinensis* spermatogenesis.

**Table 1:** **The critical DEGs involved in the “Apoptosis” pathway (pss04210) and “p53 signaling pathway” (pss04115)**.

**Gene ID**	**Read count MT_1**	**Read count MT_2**	**Read count MT_3**	**log_2_ (MT_2/MT_1)**	**log_2_ (MT_3/MT_1)**	**log_2_ (MT_3/MT_2)**	**Associated Gene Name**	**Pathway ID**
ENSPSIG00000002460	40.13793	100.46804	60.639741	1.3237	0.60602	−0.72145	PIK3CB	pss04210
ENSPSIG00000006104	65.793469	26.498994	22.200934	−1.3227	−1.5673	−0.24836	AIFM1	pss04210
ENSPSIG00000009553	721.51036	350.71682	380.07632	−1.0407	−0.91401	0.12294	PRKAR1A	pss04210
ENSPSIG00000004597	25.738277	10.178422	9.2656791	−1.3491	−1.4739	−0.12859	PIK3CD	pss04210
ENSPSIG00000018086	145.42572	631.14988	660.89061	2.1177	2.1949	0.073382	PPP3CC	pss04210
ENSPSIG00000005304	72.871495	32.992125	35.411407	−1.154	−1.0411	0.10905	IKBKB	pss04210
ENSPSIG00000016291	121.88146	42.029862	55.869293	−1.5467	−1.1254	0.41759	PPP3CB	pss04210
ENSPSIG00000004865	37.642229	22.374978	13.577431	−0.76119	−1.4711	−0.71372	CFLAR	pss04210
ENSPSIG00000010609	58.125608	44.662212	27.980516	−0.39084	−1.0548	−0.66768	PIK3R1	pss04210
ENSPSIG00000010685	28.52659	19.391648	9.1739397	−0.56759	−1.6367	−1.0729	PPP3CA	pss04210
ENSPSIG00000001225	346.54754	10.704892	6.9721941	−5.0167	−5.6246	−0.618585549	NGF	pss04210
ENSPSIG00000003288	65.528007	214.94541	388.18394	1.7245	2.5666	0.852770156	BCL2	pss04210
ENSPSIG00000003353	39.143629	183.93749	193.74099	2.2324	2.2966	0.07491366	BCL2L1	pss04210
ENSPSIG00000004168	150.08565	78.444042	26.498994	−0.936050287	−2.501776569	−1.565726282	TRADD	pss04210
ENSPSIG00000004868	17.665011	3.3343105	2.3852243	−2.4054	−2.878	−0.48326352	FADD	pss04210
ENSPSIG00000008175	28.633833	6.8441111	5.5043638	−2.0755	−2.3791	−0.314287348	MYD88	pss04210
ENSPSIG00000011998	68.152811	71.073461	158.3422	0.049815	1.2162	1.1626	APAF1	pss04210/pss04115
ENSPSIG00000016817	83.941548	66.071995	36.89153	−0.345346154	−1.186095455	−0.840749302	CASP3	pss04210/pss04115
ENSPSIG00000002892	42.028681	20.182667	11.670087	−1.0475	−1.8486	−0.790301553	CytC	pss04210/pss04115
ENSPSIG00000005528	45.310091	16.78831	11.406852	−1.4324	−2.0007	−0.5575563	FAS	pss04210/pss04115
ENSPSIG00000006057	360.91724	65.793469	29.081389	−2.455651776	−3.633500003	−1.177848227	CCNG1	pss04115
ENSPSIG00000008864	51.806561	23.076939	22.200934	−1.16668298	−1.222514457	−0.055831477	PTEN	pss04115
ENSPSIG00000009124	22.094769	115.22468	133.98664	2.3934	2.6003	0.217639323	DDB2	pss04115
ENSPSIG00000013219	23.067155	154.95769	191.6436	2.748	3.0652	0.306551424	IGF1	pss04115
ENSPSIG00000016379	14.369703	1.4916652	1.1008728	−3.268	−3.6956	−0.438276065	MDM2	pss04115
ENSPSIG00000016848	73.927738	24.270564	18.522225	−1.268	−2.0766	−0.4932	MDM4	pss04115
ENSPSIG00000015419	34.535737	13.687333	11.657537	−1.335248633	−1.566827008	−0.231578374	PERP	pss04115
ENSPSIG00000016035	71.812981	42.290165	35.921907	−0.763922461	−0.999380729	−0.235458268	SCOTIN/SHISA5	pss04115
ENSPSIG00000015871	68.420918	20.971058	33.943577	−1.7168	−1.0113	0.70169	CHEK1	pss04115
ENSPSIG00000014706	58.613263	805.58697	976.93283	3.7807	4.0697	0.28517	PPM1D	pss04115
ENSPSIG00000015497	1.0264074	31.14948	10.64177	4.9235	3.3848	−1.5425	ADGRB1	pss04115
ENSPSIG00000003194	102.58672	50.541128	31.374874	−1.0213	−1.6984	−0.6809	STEAP3	pss04115
ENSPSIG00000010284	24.849863	9.5642065	21.925716	−1.3775	−0.16989	1.2039	CDK6	pss04115
ENSPSIG00000011941	60.612056	129.77488	96.601585	1.0983	0.68317	−0.41894	RFWD2	pss04115
ENSPSIG00000013922	23.486177	5.0014658	0	−2.2421	−4.6781	−2.4398	GADD45G	pss04115
ENSPSIG00000017887	87.88549	40.362706	43.300995	−1.1333	−1.0212	0.10833	ATR	pss04115
ENSPSIG00000009470	153.04274	64.22935	109.4451	−1.2526	−0.473	0.77586	CCND3	pss04115
ENSPSIG00000003426	10.966352	3.0274001	2.94443	−1.856932487	−1.897023459	−0.040090972	SERPINB5	pss04115
ENSPSIG00000008774	117.88512	79.847962	24.633777	−0.562054184	−2.25867194	−1.696617756	PIDD1	pss04115
ENSPSIG00000009363	66.597791	43.521527	31.099655	−0.62447	−1.0986	−0.47788	RRM2	pss04115
ENSPSIG00000004513	7.6678616	19.596967	63.620459	1.4707	3.1733	1.6989	THBS1	pss04115
ENSPSIG00000012591	35.068402	26.674484	6.5134972	−0.40543	−2.4287	−2.027	CD82	pss04115

### Expression profiling of critical DEGs in *P. sinensis*

**Figure 5:**
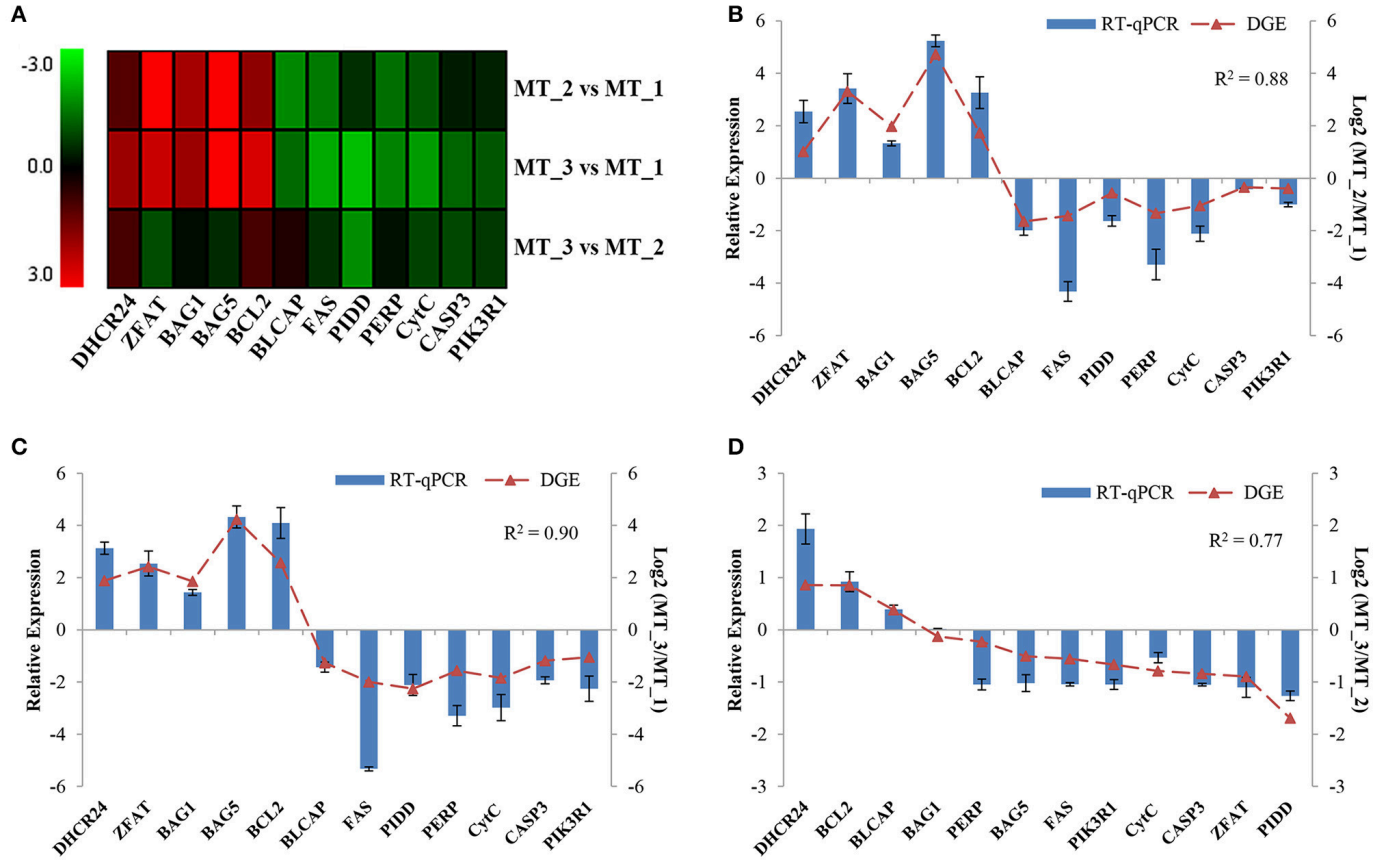
**Expression profiling of critical DEGs during spermatogenesis in ***P. sinensis***. (A)** Heat map diagrams of expression patterns of DEGs among the three libraries. The red and green bars indicate up- and down-regulated genes, respectively. The relative expression levels of DEGs between MT_2 vs. MT_1 **(B)**, MT_3 vs. MT_1 **(C)**, and MT_3 vs. MT_2 **(D)** were analyzed by RT-qPCR analysis.

To validate the dynamic expression of DEGs during spermatogenesis in *P. sinensis*, 12 genes, including *DHCR24, ZFAT, BAG1, BAG5, Bcl-2, BLCAP, FAS, PIDD, PERP, PIK3R1, CytC*, and *CASP3*, were subjected to cluster analysis of gene expression patterns (Figure 5A). Moreover, their relative expression levels were detected in the process of *P. sinensis* spermatogenesis by RT-qPCR analysis. Comparing their expression levels with the transcript abundances from RNA-Seq revealed that almost all of the selected genes shared similar expression trends between the RT-qPCR and DGE analysis in three comparisons (Figures 5B–D). To further assess the correlation between different comparisons, the correlation coefficient was calculated by SPSS software. As a result, the positive correlation between the two methods was evaluated in the comparisons of MT_2 vs. MT_1, MT_3 vs. MT_1, and MT_3 vs. MT_2 with *R*^2^ = 0.88, 0.90, and 0.77, respectively, identifying the reliability and accuracy of the RNA-Seq data.

### Measurement of mitochondrial transmembrane potential

**Figure 6:**
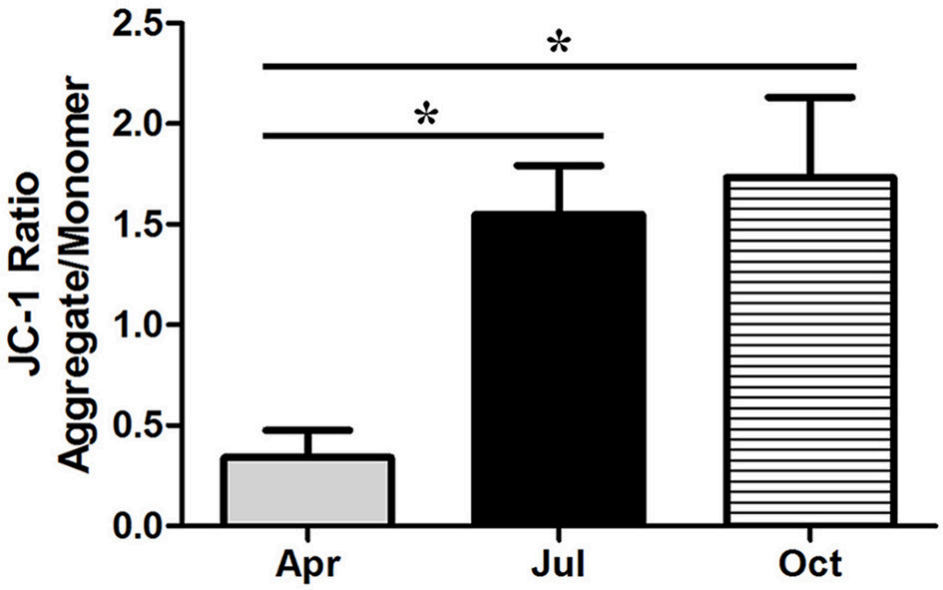
**Changes in mitochondrial transmembrane potential in ***P. sinensis*** testis**. The values (^*^) obtained in July and October were significantly higher (*P* < 0.05) than those obtained in April.

The disruption of mitochondrial transmembrane potential is an early step in the apoptotic process. In this study, to further assess the apoptosis of *P. sinensis* testis during spermatogenesis, the changes in the mitochondrial transmembrane potential were monitored by JC-1 dye. As shown in Figure 6, the mitochondrial transmembrane potential of the isolated mitochondria was significantly lower (*P* < 0.05) in April than in July and October, which is in agreement with the higher ratio of apoptosis in April by TUNEL analysis.

### Expression of Bcl-2, Cleaved caspase-3 and CytC proteins in the *P. sinensis* testis during spermatogenesis

**Figure 7:**
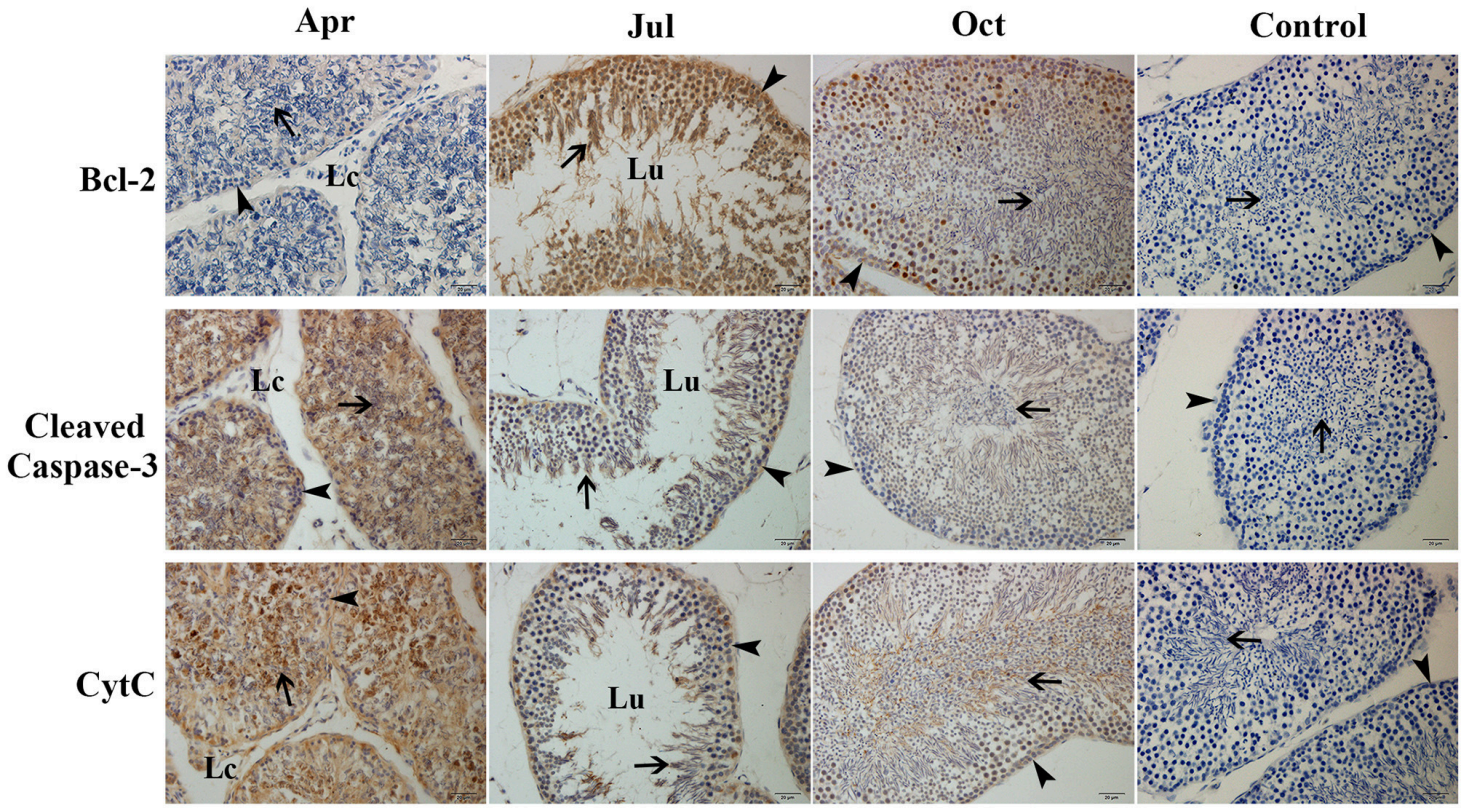
**Immunostaining of Bcl-2, Cleaved Caspase-3, and CytC in the testis of ***P. sinensis*** during spermatogenesis**. Arrowhead, Spermatogonia; arrow, spermatozoa; Lc, Leydig cell; Lu, lumen. Scale bar = 20 μm.

To investigate the dynamic expression of critical proteins related to apoptosis during spermatogenesis, IHC analysis was used to observe the positive reaction of Bcl-2, Cleaved caspase-3, and CytC proteins in the *P. sinensis* testis (Figure 7). In April, the germ cells in the seminiferous epithelium showed weak immunoreactivity of the Bcl-2, whereas strong immunoreactivity of Cleaved caspase-3 and CytC was observed. In July and October (intermediate and late spermatogenesis), germ cells displayed intense Bcl-2 immunostaining, while moderate immunoreactivity of the Cleaved caspase-3 and CytC was observed. No staining was detected in the negative control sections.

**Figure 8:**
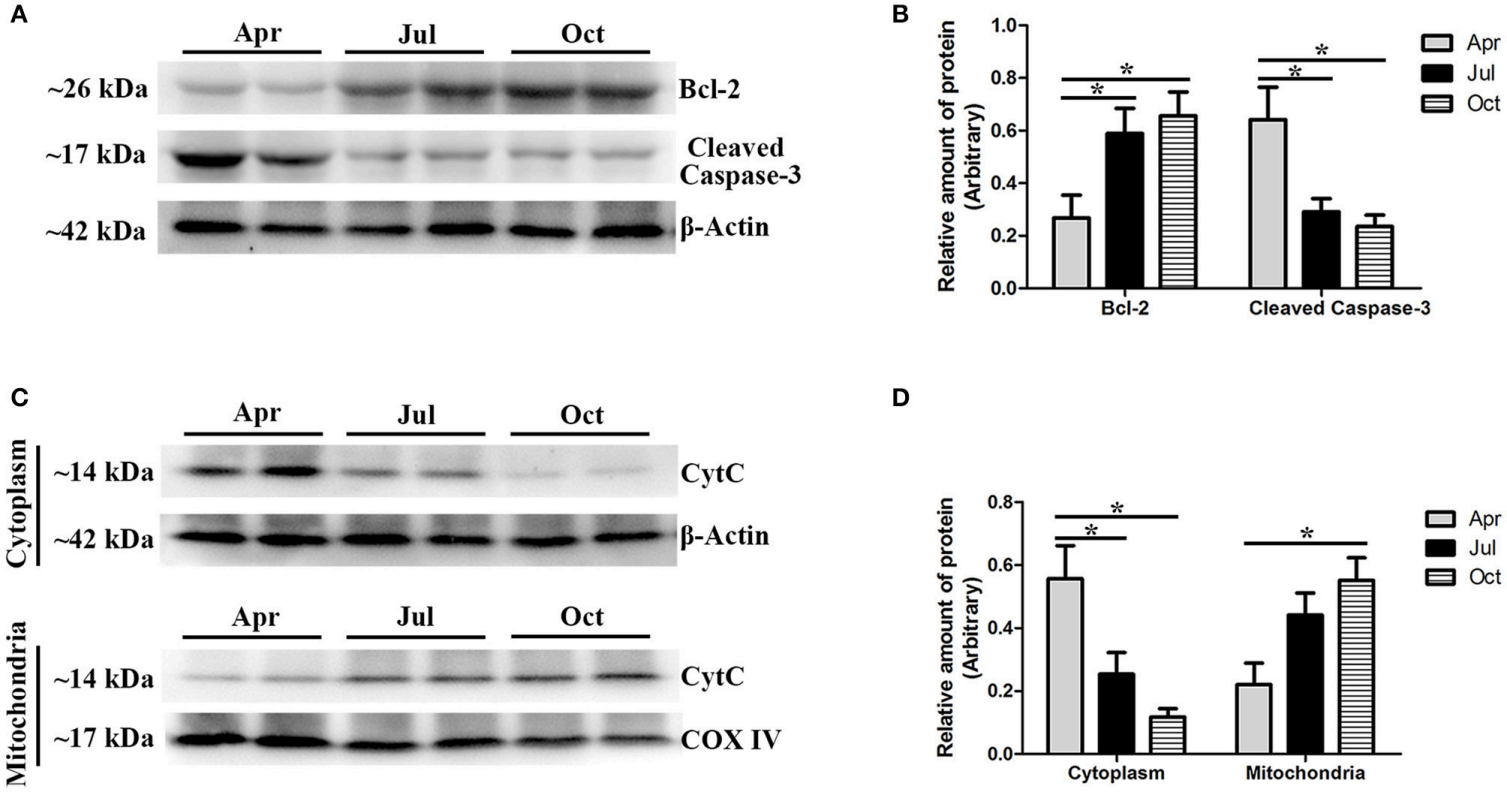
**Western blot analysis of Bcl-2, Cleaved Caspase-3, and CytC protein expression in the testis of ***P. sinensis*** during spermatogenesis. (A,B)** The protein expression levels of Bcl-2 and Cleaved Caspase-3. **(C,D)** The protein expression level of CytC. The histogram represents the densitometric analysis of the immunoblots. ^*^P < 0.05 compared to April.

Moreover, western blot analysis was performed to further validate the relative protein levels of Bcl-2 and Cleaved caspase-3 in *P. sinensis* testis, with β-Actin as an internal control (Figures 8A,B). As shown in Figure 8A, immunopositive protein bands for the Bcl-2 and Cleaved caspase-3 forms were clear and evident in each sample. The level of Bcl-2 protein was significantly increased (*P* < 0.05) in July and October compared with that in April, while the Cleaved caspase-3 protein was significantly decreased (*P* < 0.05), indicating that the apoptosis was down regulated in the period of intermediate and late spermatogenesis (Figure 8B). Furthermore, to evaluate the effect of seasonal change on the release of pro-apoptotic protein CytC from the mitochondria in *P. sinensis* testis, the protein extracts of both the mitochondrial and cytosolic fractions were used for western blot analysis (Figures 8C,D). The results showed that the CytC content in the cytoplasm of *P. sinensis* testis was significantly reduced (*P* < 0.05) from April to October, whereas the amount in the mitochondria was gradually increased (*P* < 0.05). Moreover, between July and October, the CytC content showed no significant changes (*P* > 0.05) in the cytoplasm or mitochondria. These findings imply that the variation of the CytC protein might directly represent the occurrence of apoptosis during spermatogenesis in *P. sinensis*.

## Discussion

Spermatogenesis within the Chinese soft-shelled turtle (*Pelodiscus sinensis*) is closely parallel to that seen in most temperate and boreal reptilian species (Rheubert et al., 2009b; Sousa et al., 2014). As the germ cells developed spermatogenically, *P. sinensis* spermatogenesis initiated from the early spring, continued through the summer, and was completed by autumn, which was similar to the process described in other temperate-zone turtles such as *Trachemys scripta, Apalone ferox*, and *Graptemys pseudogeographica kohnii* (Meylan et al., 2002; Gribbins et al., 2003; Lancaster et al., 2015). In ground skink (*Scincella lateralis*), spermatogenesis following a dissociated reproductive pattern ceased in autumn, and a massive spermiation event from the seminiferous tubules to the epididymal canals was performed to keep the spermatozoa until the next breeding season (Rheubert et al., 2009a). Furthermore, Gribbins et al. summarized a similar process in the black swamp snake (*Seminatrix pygaea*) as a postnuptial pattern of development in which sperm development began immediately after mating and continued through the months of breeding (Gribbins et al., 2005). In this current study, morphological evidence clearly confirmed that the sperm development strategy of *P. sinensis* belonged to a dissociated rather than associated pattern and seemed to follow the postnuptial pattern, which was consistent with our previous research (Zhang et al., 2007, 2008). This male gamete production pattern was different from the consistent spatial development strategy among temperate-zone breeding birds and mammals and may represent an evolutionarily intermediate stage with respect to amniotic germ cell development.

Apoptosis is a highly regulated and conserved mechanism that plays a pivotal role in the process of gamete maturation and embryogenesis (Brill et al., 1999). A large number of studies have indicated that apoptosis, as the regulatory mechanism of male germ cell development, is implicated in spermatogenesis and plays a critical role in eliminating the germ cells that are defective or carry DNA mutations (Rodriguez et al., 1997; Russell et al., 2001; Schaller et al., 2008). Previous studies have examined the complex changes that occur in testicular cells during seasonal reproductive regression, highlighting that apoptosis might be the contributing mechanism of testicular atrophy in most seasonally breeding species (Young and Nelson, 2001). However, other studies have reviewed the role of apoptosis in the testicular regression of some species. For instance, in both roe deer and Iberian mole, apoptosis was not responsible for massive germ-cell depletion at any of the reproductive stages (Blottner et al., 2007; Dadhich et al., 2010). In the present study, TUNEL staining, a routine and normal method for visualizing cells undergoing apoptosis, showed that more apoptotic cells were observed in April (the spermatogenically quiescent phase) than in July and October (intermediate and later spermatogenesis). These observations were consistent with our previous studies in which the apoptosis remained at a low level throughout the active phase of *P. sinensis* spermatogenesis (Zhang et al., 2008). Strikingly, positive staining was observed most frequently in spermatids and spermatozoa during the spermatogenically quiescent phase, and typical apoptotic morphology was also demonstrated in TEM samples. Our H&E staining and previous studies demonstrated that the germ cells of male *P. sinensis* progressed through the process of spermatogenesis as a single cohort, leading to one spermiation event (Zhang et al., 2015; Nisar et al., 2016). Indeed, the residual spermatids and spermatozoa in the seminiferous tubules needed to be eliminated, which was an essential program for the next spermatogenesis cycle in the Chinese soft-shelled turtle (Zhang et al., 2007). Fortunately, our evidence has obviously shown that most of the residual spermatids and spermatozoa underwent apoptosis during the spermatogenically quiescent phase, implying that apoptosis may be employed as one of the underlying mechanisms to clear the degraded germ cells. These results have indicated that apoptosis may be responsible for seasonal variations in the spermatogenesis of *P. sinensis*.

It has been extensively studied that cell death by apoptosis is exerted by the coordinated action of many different genes (Müllauer et al., 2001). Recently, the NGS-based RNA-Seq approach has been widely employed for the identification and profiling of apoptosis-related genes in many species (Deng et al., 2014; Liu et al., 2015; Yue et al., 2015). In this study, using RNA-Seq technology, many DEGs involved in apoptosis regulation were identified and characterized at the different stages of spermatogenesis in *P. sinensis*. To the best of our knowledge, this is the first report on the systematic and comprehensive profiling of apoptosis-related genes during spermatogenesis in reptiles. Remarkably, during intermediate and late spermatogenesis (in MT_2 and MT_3 libraries), some anti-apoptosis and pro-apoptosis genes were significantly differentially expressed and showed up-regulated and down-regulated patterns, respectively. Among these anti-apoptosis genes, *DHCR24*, encoding the 3β-hydroxysterol Δ24-reductase, a member of the flavin adenine dinucleotide-dependent oxidoreductase, has been reported to possess anti-apoptotic activity toward many types of cells (Di et al., 2005). Functional studies have demonstrated its effect in protecting cells from oxidative stress-induced apoptosis, which was coordinated with lower caspase-3 activity (Lu et al., 2008). Another anti-apoptotic molecule, ZFAT, also known as ZNF406, encoding a zinc-finger protein, is highly conserved among species and has been reported to be a critical molecule for cell survival through regulating the pathways of Bcl-2 and IL-6-mediated apoptosis in mouse embryonic fibroblasts (Fujimoto et al., 2009; Doi et al., 2015). In agreement with these previous reports, our studies exhibited the increased expression of *PsDHCR24* and *PsZFAT* in MT_2 and MT_3 libraries and implied their negative roles in apoptosis during intermediate and late spermatogenesis in *P. sinensis*.

It is reported that BAG1, belonging to the Bcl-2-associated athanogene (BAG) family, not only inhibited apoptosis independently but also through interaction with Bcl-2 (Takayama et al., 1995). A previous study has shown that the increased expression of BAG1 contributes to enhancing the resistance of cervical cells against apoptosis induced by a DNA-damaging reagent (Chen et al., 2002). Moreover, BAG5 as a unique member of the BAG family, contains five BAG domains and was also reported to share the function of anti-apoptotic activity (Bruchmann et al., 2013). Ma et al. suggested that BAG5 could inhibit apoptosis through both endogenous and mitochondria-mediated pathways in PC12 cells (Ma et al., 2013). In this study, the increased mRNA levels of *BAG1, BAG5*, and *Bcl-2* were detected by both the Clustering and RT-qPCR analysis during intermediate and late spermatogenesis in *P. sinensis*. Moreover, IHC and western blot analysis further revealed the high level of Bcl-2 protein in the *P. sinensis* testis in July and October. Numerous studies have suggested the non-redundant and lineage-specific roles of Bcl-2 in preventing germ cell death during spermatogenesis. For instance, in chicken, Bcl-2 was highly expressed in spermatogonia during spermatogenesis and had the ability to block apoptosis for spermatogonia (Vilagrasa et al., 1997). Furuchi *et al*. reported that the low levels of Bcl-2 in germ cells would accelerate the apoptosis of mature germ cells and result in the subsequent disruption of spermatogenesis (Furuchi et al., 1996). Additionally, a pro-apoptosis molecule, BLCAP, which was down-regulated in our results, has been reported to trigger apoptosis by inactivating anti-apoptosis Bcl-2 protein in human Ewing's sarcoma cells, and its overexpression could exhibit the inhibition of cell growth by inducing apoptosis and S-phase arrest in human cancer cells (Yao et al., 2007; Fan et al., 2011). In general, the differential expression of these apoptosis-related genes may contribute to inhibiting apoptosis in the testes and prevent germ cell loss at the spermatogenically active phase of *P. sinensis*.

The p53 signaling pathway has been shown to mediate a variety of intrinsic and extrinsic stress responses including apoptosis in various cells (Clarke et al., 1993). It was demonstrated that the p53 signaling pathway played critical functions in germ cell apoptosis in both mammalian and reptilian species, as it could regulate the expression of not only apoptosis-related proteins such as Bax, Bid, Bcl-2, and Bcl-xL but also death receptors such as CD95, FAS, Apo-1, and DR5 (Beumer et al., 1998; Allemand et al., 1999; Laptenko and Prives, 2006; Vousden and Prives, 2009). Previous studies have reported that p53-mediated apoptosis was responsible for the initial phase of germ cell apoptosis during spermatogenesis in cryptorchid mice (Yin et al., 1998a,b). Furthermore, under genotoxic stress, the p53 signaling pathway was involved in the apoptosis of spermatogonia, spermatocytes, and spermatids (Hasegawa et al., 1998; Allemand et al., 1999). In this study, the expression levels of several transcripts such as *FAS, PIDD, PERP, CytC*, and *CASP3*, which are implicated in the p53 signaling pathway, were significantly decreased in July and October, implying that p53 cascades contributed to repressing the apoptotic effect in the testis and protecting the germ cells lost during intermediate and late spermatogenesis in *P. sinensis*. CytC is a soluble protein and is loosely bound to the outer face of the inner mitochondrial membrane (Cai and Jones, 1998). Owing to the depolarization of mitochondrial membrane potential in apoptotic cells, the mitochondrial permeability transition pore opened and CytC was released from the mitochondria into the cytoplasm. Together with Apaf-1 and procaspase-9, CytC was assembled into the apoptosome to activate caspase-9, which further activated effector caspase-3 to induce cell death (Jiang and Wang, 2004). In this study, JC-1 staining revealed a rapid increment of the mitochondrial transmembrane potential in July and October. Moreover, the results of western blot analysis showed low protein levels of pro-apoptotic cleaved caspase-3 and CytC in the cytoplasm, which was further confirmed by IHC analysis. These observations were consistent with previous studies in various mammalian cells. Indeed, the CytC-Caspase axis in the p53 signaling pathway has been suggested to play a pivotal role in the execution of apoptosis under various physiological and pathological conditions (Zhang et al., 2014; Yang et al., 2015). Taken together, it is reasonable to infer that the seasonal effects of germ cell apoptosis might be monitored by the CytC-Caspase model involved in the p53 signaling pathway during spermatogenesis in *P. sinensis*.

In summary, this study validated the dynamic changes that occur in apoptosis during spermatogenesis and described the characterization of apoptotic cells in the testes of *P. sinensis* through various morphological studies. These findings revealed that apoptosis might be the key mechanism underlying seasonal spermatogenesis and function in the degraded germ cells of *P. sinensis* testes. Moreover, the identification and expression profiling of apoptosis-related genes were performed by RNA-Seq and DGE analysis, which, combined with the analysis of critical protein levels, indicated that the CytC-Caspase regulatory model may determine the variations of apoptosis during spermatogenesis in *P. sinensis*.

## Author contributions

The authors have made the following declarations about their contributions: TL and QC conceived and designed the experiments. TL, LW, HC, YH, PY, and NA performed the experiments. TL, TW, and YL analyzed the data. TL and QC wrote the paper. All authors read and approved the final manuscript.

## Funding

This research was supported by grants from the National Natural Science Foundation of China (Grant number: 31672505) and Priority Academic Program Development of Jiangsu Higher Education Institutions, PR-China.

### Conflict of interest statement

The authors declare that the research was conducted in the absence of any commercial or financial relationships that could be construed as a potential conflict of interest.
