# Supplementary material for: Molecular and Cellular Mechanisms of Apoptosis during Dissociated Spermatogenesis
Source: Front Physiol. 2017 Mar 29;8:188. doi: 10.3389/fphys.2017.00188 (PMC5372796; doi:10.3389/fphys.2017.00188)
Supplement: Supplementary file 1 [file Table1.DOCX]

**Supplementary Table 1** Primers of critical DEGs for RT-qPCR analysis in *P. sinensis*

| **Gene name** | **Sense primer (5'-3')** | **Antisense primer (5'-3')** |
| --- | --- | --- |
| DHCR24 | GTCTTCGTCTGTCTCTTC | CCACCTTCATTCTTCCAT |
| ZFAT | TCAGAAGCACAGTAACATT | ACAGACAGGACAGGAATA |
| BAG1 | CCTGGCTACAATGAAGTT | TGACAATATCCTGGTATGC |
| BAG5 | CACATCCTTCAGTCTCAA | TAATCTCTTCCACCACTTC |
| BCL2 | GCAGTAATGGATACAGACA | GAAGAGGAGGAGGAGTTA |
| BLCAP | TCTTGACTCTGGATACGA | ACATTAACAGTGGTTGGT |
| CytC | GATTCTCTTACTCGGATTCA | CTGCTCTCTCATTCTTCTT |
| FAS | ATACACGGAACAAGACAAT | CCACTAAGAGGAGAATTATGATA |
| PIDD | AACATTCCTCATCTCATCTC | TTACATCAACCACCAGAAG |
| PERP | CCAACTACGAAGATGAGAT | TCTATTCTATAACTCCAACACTA |
| CASP3 | GCATTGAGACAGACAGTA | CGCCAAGAGTAATAACCTA |
| PIK3R1 | TGGTGGAAGATGATGAAG | TGTGTTGGTAATGTAGCA |
| β-Actin | AGACCCGACAGACTACCTCA | CACCTGACCATCAGGCAACT |
