# Supplementary material for: Molecular and Cellular Mechanisms of Apoptosis during Dissociated Spermatogenesis
Source: Front Physiol. 2017 Mar 29;8:188. doi: 10.3389/fphys.2017.00188 (PMC5372796; doi:10.3389/fphys.2017.00188)
Supplement: Supplementary file 2 [file Table2.DOCX]

**Supplementary Table 2** Summary of read numbers in the MT_1, MT_2 and MT_3 libraries based on the *P. sinensis* reference genome

| **Sample name** | **MT_1** | **MT_2** | **MT_3** |
| --- | --- | --- | --- |
| Raw reads | 65,373,182 | 46,716,990 | 44,988,350 |
| Clean reads | 62,840,880 | 45,060,196 | 43,378,150 |
| Total mapped | 44,810,353 (71.31%) | 32,955,437 (73.14%) | 31,859,358 (73.45%) |
| Multiple mapped | 1,120,849 (1.78%) | 943,251 (2.09%) | 947,107 (2.18%) |
| Uniquely mapped | 43,689,504 (69.52%) | 32,012,186 (71.04%) | 30,912,251 (71.26%) |
| Reads map to '+' | 21,870,130 (34.8%) | 15,988,072 (35.48%) | 15,433,257 (35.58%) |
| Reads map to '-' | 21,819,374 (34.72%) | 16,024,114 (35.56%) | 15,478,994 (35.68%) |
| Non-splice reads | 28,322,324 (45.07%) | 19,734,652 (43.8%) | 19,266,549 (44.42%) |
| Splice reads | 15,367,180 (24.45%) | 12,277,534 (27.25%) | 11,645,702 (26.85%) |
